# Lymphoma with tuberculous granulomas

**DOI:** 10.1002/ccr3.5431

**Published:** 2022-02-09

**Authors:** Jyoti Mohan Lal, Anila Rashid

**Affiliations:** ^1^ 66705 Section of Hematology & Transfusion Medicine Department of Pathology and Laboratory Medicine Aga Khan University Hospital Karachi Pakistan; ^2^ 66705 Section of Hematology & Transfusion Medicine Department of Pathology and Laboratory Medicine/Oncology Aga Khan University Hospital Karachi Pakistan

**Keywords:** hematology, infectious diseases, oncology

## Abstract

Chronic granulomatous inflammation is a common finding in lymphoproliferative disorders (LPDs), but it is important to exclude coexisting mycobacterium tuberculosis (MTB) especially in patients from areas of high endemicity. This case emphasizes the relevance of performing MTB culture on bone marrow exhibiting LPD and concomitant granulomas.

## Graphical abstract

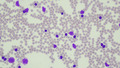

## 1 CLINICAL IMAGES

**FIGURE 1:**
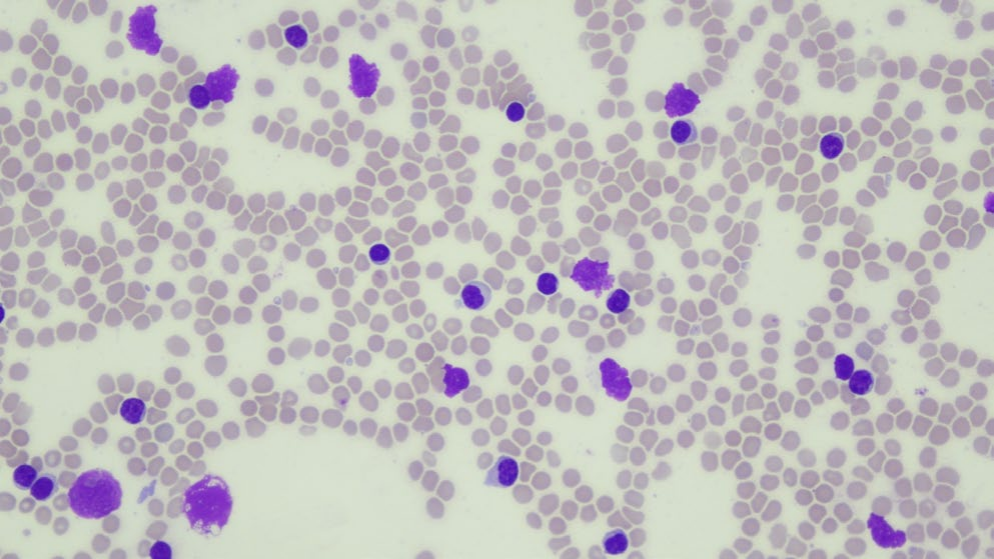
Peripheral blood smear at 40× showing lymphocytosis and smudge cells

**FIGURE 2:**
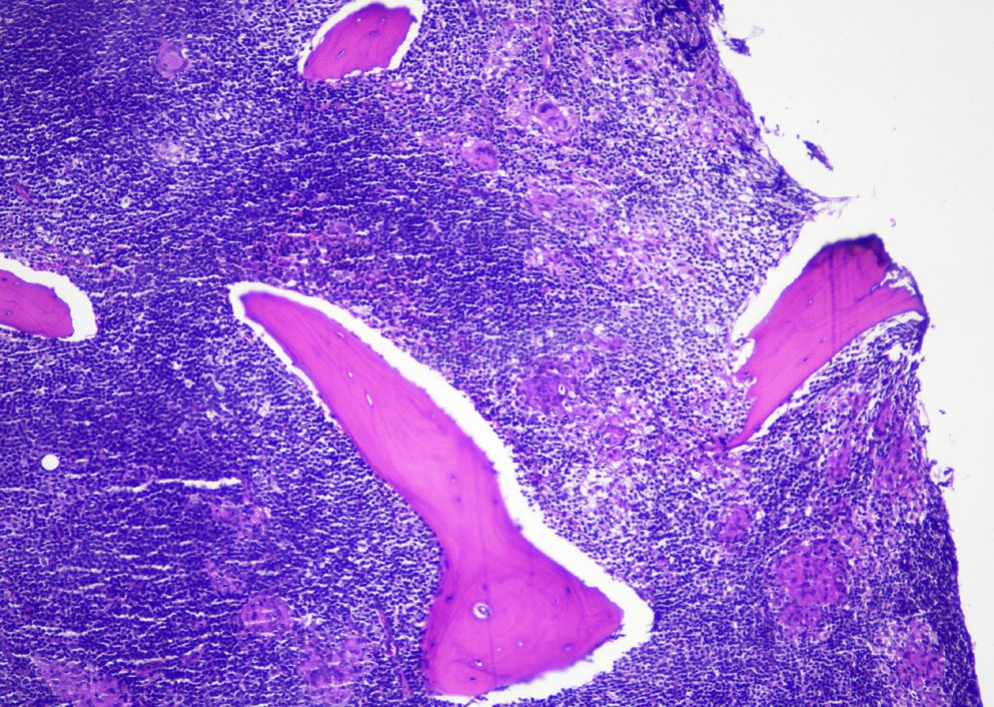
Bone trephine H&E section at 10× exhibiting diffuse lymphoid cell infiltration with multiple granulomas and giant cells

**FIGURE 3:**
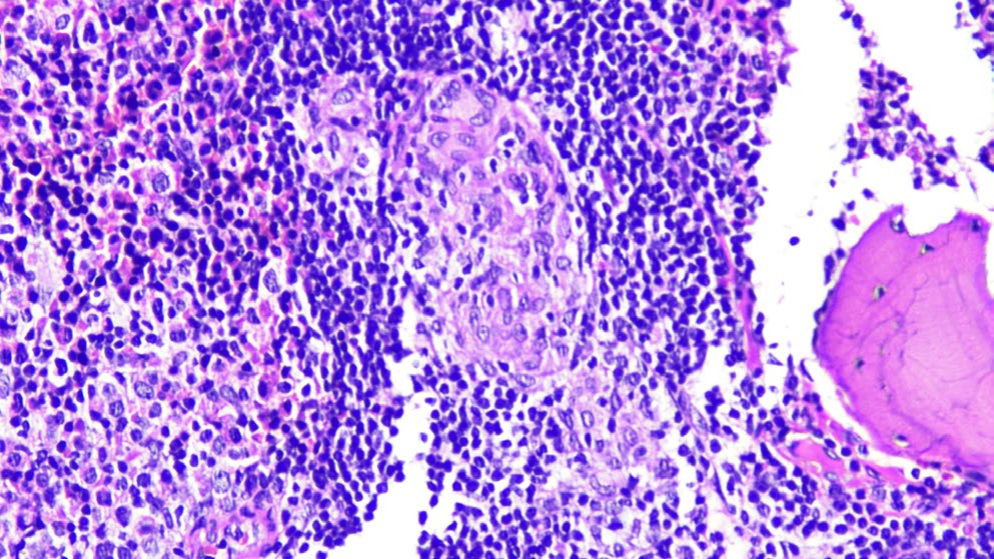
Granuloma at 40×

**FIGURE 4:**
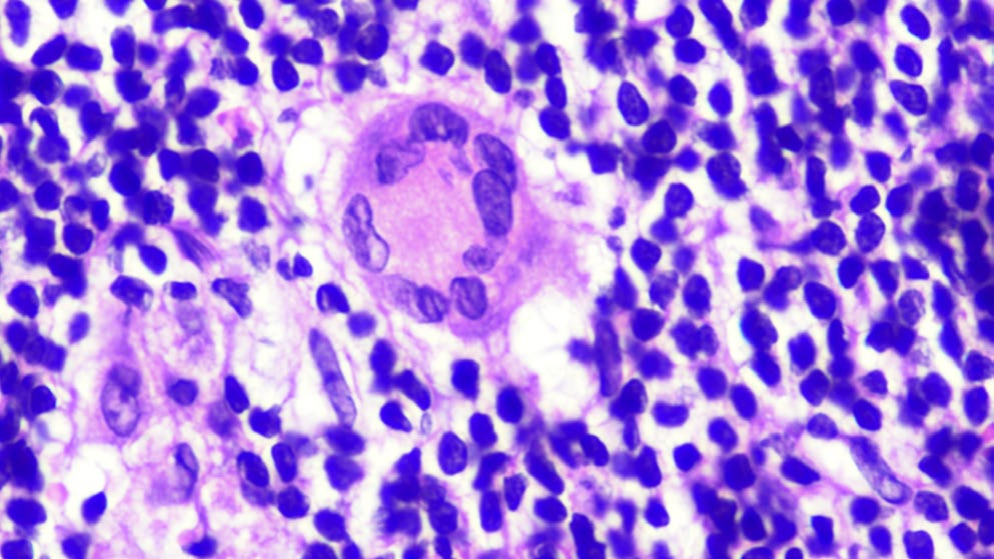
Langhan's giant cell at 100×

**FIGURE 5:**
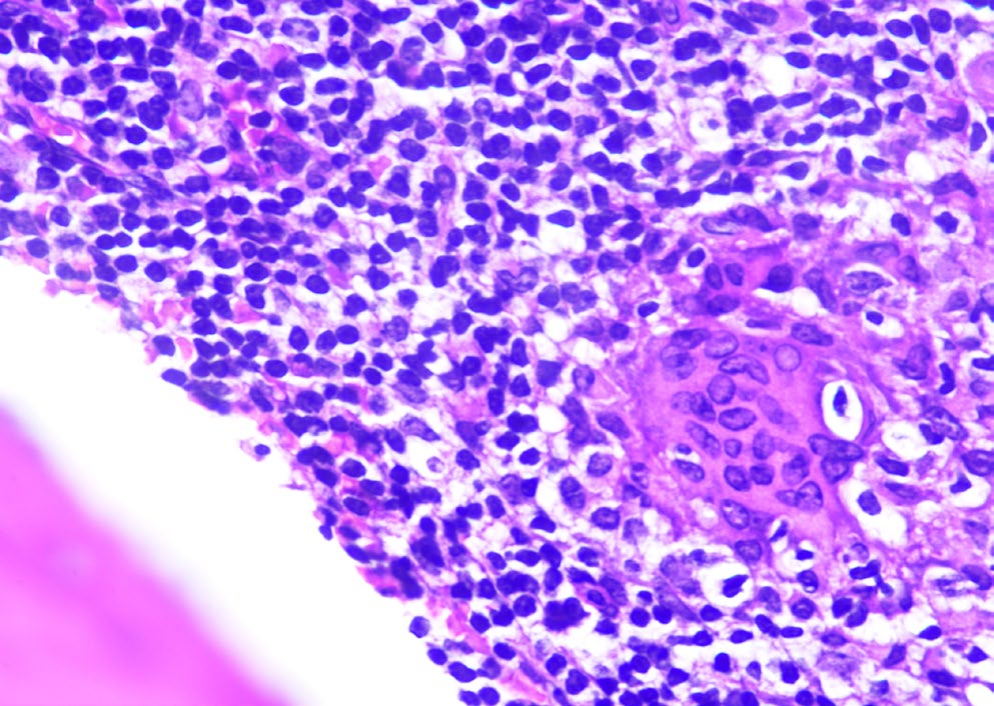
Foreign‐body giant cell at 100×

**FIGURE 6:**
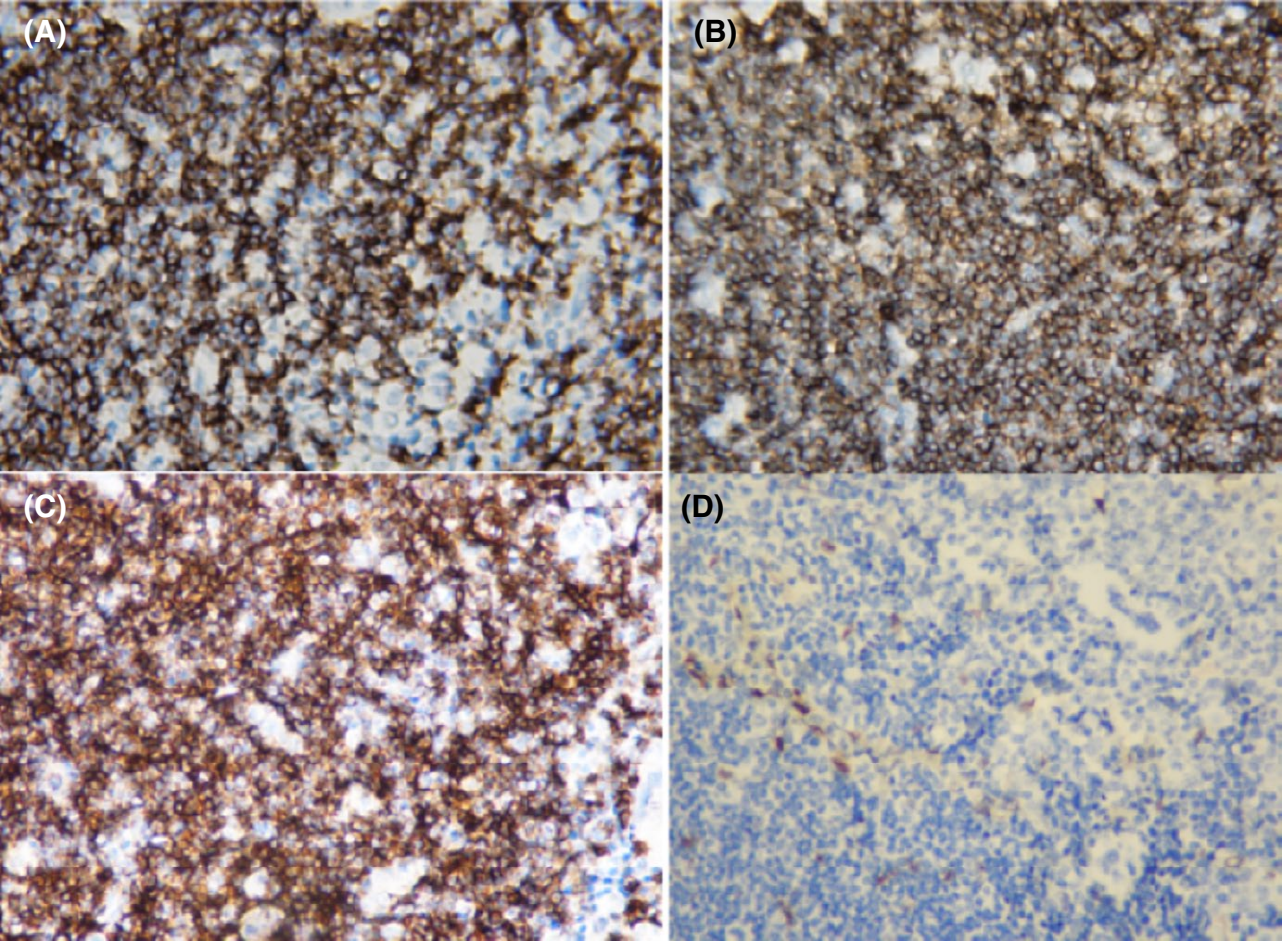
Immunohistochemistry panel showing lymphoid cells positive for CD20 (A), CD5 (B), CD23 (C), and negative for CD3 (D)

A 40‐year‐old woman presented with a 5‐month history of easy fatigability and weight loss. Examination revealed cervical lymphadenopathy and splenomegaly. Radiological scan revealed abdominal and pelvic lymphadenopathy, and splenomegaly. Complete blood count showed Hb:11.8 g/dl, HCT:37%, WBC:38 × 10^9^/L, platelets: 193 × 10^9^/L. Blood smear revealed normocytic normochromic red blood cells, lymphocytosis (83%), and smudge cells (Figure 1). Bone marrow exhibited diffuse infiltration with small lymphoid cells and multiple areas of chronic granulomatous inflammation composed of histiocytes, epithelioid cells, and multinucleated Langhan's type and foreign‐body giant cells (Figures 2, 3, 4, 5). Immunohistochemistry panel was performed that showed positive CD20, CD5, CD23, and Bcl2 and negative CD3, CD10, Bcl6, cyclin D1, and Ziehl–Neelsen stain (Figure 6). The diagnosis of chronic B‐cell lymphocytic leukemia was made with co‐existing chronic granulomatous inflammation. As lymph node culture was negative, bone marrow aspirate was sent for mycobacterial tuberculosis (MTB) culture (LJ and MGIT) and acid‐fast bacilli (AFB) smear, which showed MTB growth with negative smear. In our facility, a solid and a liquid culture media are used for MTB diagnosis irrespective of smear results.

This case highlights the importance of considering tuberculosis in patients with granulomas in hematolymphoid malignancies from areas of high endemicity.^1^, ^2^ In cases, where lymph node MTB culture is not helpful, bone marrow aspirate should be sent for MTB culture.

## CONFLICT OF INTEREST

None.

## AUTHOR CONTRIBUTIONS

JLM took images and drafted the manuscript. AR conceived the idea, drafted, and critically reviewed the manuscript.

## ETHICAL APPROVAL

Institutional ethics approval was obtained.

## CONSENT

Written informed consent was obtained from the patient to publish this report in accordance with the journal's patient consent policy.

## Data Availability

Data sharing is not applicable to this article as no new data were created or analyzed in this study.
